# Editorial: Intelligent neural interface for healthcare and rehabilitation

**DOI:** 10.3389/fbioe.2024.1406620

**Published:** 2024-04-16

**Authors:** Tianzhe Bao, Yingchun Zhang, Zhi-Qiang Zhang, Ping Zhou

**Affiliations:** ^1^ School of Rehabilitation Science and Engineering, University of Health and Rehabilitation Sciences, Qingdao, China; ^2^ Department of Biomedical Engineering, University of Miami, Coral Gables, FL, United States; ^3^ School of Electronic and Electrical Engineering, University of Leeds, Leeds, United Kingdom

**Keywords:** neural interface, healthcare monitoring, rehabilitation training, robotic control, human-machine interaction

The goal of intelligent neural interface (NI) is to forge a direct line of communication between our neural systems and external gadgets, holding a strong potential to markedly enhance life quality of elderly and disabled people. This Research Topic has collected a group of articles that provide innovative methods and perspectives in bioelectronics, biomechanics, biomaterials, and biosignal analysis, etc., to promote the development and application of human-machine interaction (HMI) in both healthcare and rehabilitation.

A main role of NI is the enhancement of robot-assistant rehabilitation to improve motor function of physically and neurologically impaired patients. However, inappropriate parameters in assist-as-needed (AAN) control strategy can lead to unstable and oscillating robot behaviors. To address this issue, Xu et al. introduced a control framework to simultaneously adjust both the training task and robotic assistance in a lower extremity rehabilitation robot according to the subjects’ performances based on EMG analysis. A trajectory deformation algorithm was developed to plan the robot’s desired trajectory as the high-level controller, whilst an AAN control strategy with a feedback gain modification algorithm was employed to regulate the robotic assistance as the low-level controller. Both the training task and robotic assistance adaptation algorithms were then integrated into a framework to generating a smooth and compliant desired trajectory. This protocol offers an avenue to enable human-in-the-loop optimization in robot-assisted rehabilitation.

Characteristics of electrodes are critical for information transmission between external electrical circuit and internal peripheral nervous system. To establish a better pathway, Tang et al. introduced the innovative design of microneedle array electrodes (MAEs) by using a magnetization-induced self-assembly method. The developed MAEs showed significantly lower and more stable electrode-skin interface impedance than the metal-based flat array electrodes (FAEs), resulting in a notable increase in signal-to-noise ratio (SNR) of more than 30% and a consequential 10% improvement in motion-intention classification. The feasibility of MAE-based electrical stimulation for sensory feedback was verified through electroencephalography (EEG) analysis. Further enhancements to the structural strength of the microneedles were also suggested to ensure long-term reliability.

Flexible tactile sensors offer another important monitoring pathway in NI. Liu et al. extensively discussed the materials and preparation technologies of flexible tactile sensors, and summarized various applications in human signal monitoring and robotic tactile sensing. In particular, traditional HMI mainly concentrates on the control of robot/machine but lacks sufficient feedback to humans, whereas there are now increasing demands for intuitive and effective manipulation via closed-loop control using flexible tactile sensors, such as the application of tactile feedback glove in virtual reality. This review provides us a new perspective on the pivotal role of flexible tactile sensors in advancing intelligent HMI.

In the context of rehabilitation training, the recovery of gait function is of great significance. To break the limitation in functional electrical stimulation (FES) in foot drop correction, Dong et al. developed a timing- and intensity-adaptive FES control strategy for both the tibialis anterior (TA) and gastrocnemius (GAS). The designed hybrid control strategy signifies a departure from traditional FES methods by incorporating both a speed-adaptive (SA) module for controlling stimulation timing, and an iterative learning control (ILC) module for regulating stimulation intensity based on real-time kinematic or kinetic data. It is noted that the presented design not only improved the angles of ankle and knee joints of patients after stroke, but also increased anterior ground reaction force. By addressing the less-explored but equally affected GAS muscle during the stance phase, a more comprehensive treatment can be realized.

Apart from FES, neuromuscular electrical stimulation (NMES) has also been widely employed to stimulate activity-dependent plasticity in brain circuitry. Cui et al. explored the cortical activation patterns induced by NMES when synchronized with rehabilitation strategies in mirror neuron system (MNS). Based on three novel rehabilitative treatments, i.e., action observation (AO), action execution (AE), and action imitation (AI), the feasibility of synchronous application of NMES and mirror neuron rehabilitation was experimentally verified. This study contributes valuable insights and as well empirical evidence that can inform the design, implementation, and optimization of neurofeedback therapy rehabilitation devices.

Considering that the non-stationary characteristics of biosignals poses challenges to real-time application of NI, Wang et al. discussed the calibration burdens caused by data distribution shift. Several representative scenarios, including electrode shift, cross-user, cross-day, cross-set, and unwanted action interference are investigated. State-of-the-art approaches that improve the robustness of myoelectric control were systematically introduced, mainly classified into three categories, i.e., data manipulates, feature manipulates, and model structure. An important aspect highlighted by the study is the significance and feasibility of embedding musculoskeletal information in data-driven framework to deal with EMG variations. This underscores a trend towards leveraging machine/deep learning and physical/physiological knowledge in the development of more interpretable, trustworthy, and generalizable motion-intention estimators.

Despite aforementioned discoveries and technical advances, several challenges warrant further attention to foster more reliable and broadly applicable NI systems. Firstly, there is a pressing need to substantially augment the availability of high-quality public datasets tailored for the development and validation of deep learning techniques, and potentially, NI-specific “large models.” Unlike traditional physical data types such as images, videos, and audio, physiological data exhibit nuanced non-structural characteristics, intensifying the complexity of analysis while underscoring its paramount importance. Given the inherent limitations in information density across current sensing modalities, multi-modal fusion is also of significance to bolster the accuracy and resilience of either motion estimation or health assessments. Additionally, energy consumption should be considered to enable better wearability and accessibility of NI. Therefore, low cost and energy-efficient hardware are highly urged.

We hope that this Research Topic will promote more interesting thoughts and perspectives on NI-based intelligent healthcare and rehabilitation, and help to spark a rich dialogue among researchers, clinicians, and technologists in a broader view of HMI.

